# Development of *in vitro* potency assays for AAV-based gene silencing therapies targeting FSHD and CMT1A

**DOI:** 10.1016/j.omta.2026.201727

**Published:** 2026-04-01

**Authors:** Jason McCoy, Lindsay M. Wallace, Bi Zhou, Brian Price, Rachel Salzman, Scott Q. Harper

**Affiliations:** 1 Jerry R Mendell Center for Gene Therapy, The Abigail Wexner Research Institute at Nationwide Children’s Hospital, Columbus 43210, OH, USA; 2 Department of Pediatrics, The Ohio State University College of Medicine, Columbus 43210, OH, USA; 3 ArmatusBio Inc, Columbus 43210, OH, USA

**Keywords:** potency assay, RNAi, miRNA, gene silencing, AAV, FSHD, CMT1A, AAV receptor, luciferase assay

## Abstract

Adeno-associated viral (AAV) vectors are a cornerstone system for delivering gene therapies for several diseases, including two under development in our lab: Charcot-Marie-Tooth disease type 1A (CMT1A) and facioscapulohumeral muscular dystrophy (FSHD). Although most AAV therapies today involve gene replacement for recessive disorders, CMT1A and FSHD are dominant diseases that would benefit from disease gene silencing, and we have generated extensive pre-clinical safety and efficacy data to support translating gene therapies for both diseases. Here, in anticipation of clinical trials and, optimistically, post-approval, we describe our approach to develop a robust potency assay to assess product strength and stability. To do this, we modified HEK293T cells to increase permissibility to AAV transduction and produce a quantifiable, treatment-responsive readout. Specifically, we created stable cell lines containing (1) the AAV receptor (AAVR) to improve AAV transduction and (2) a *Renilla* luciferase (*rLuc*) open reading frame with disease gene sequences in the 3′ UTR, to enable disease gene knockdown quantification by luciferase assay. Our study provides a straightforward framework for potency assay development supporting AAV-mediated and non-viral gene silencing programs.

## Graphical abstract

**Figure:**
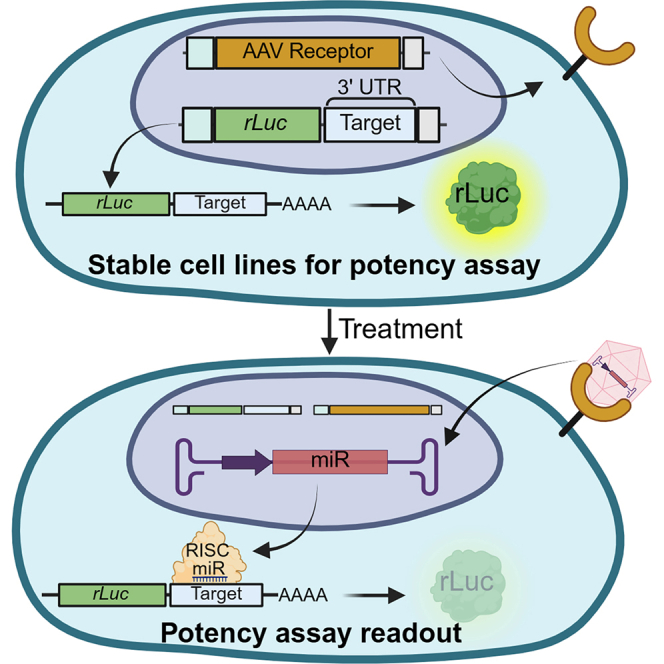

## Teaser

This study describes *in vitro* potency assays to support AAV-based gene silencing programs for FSHD and CMT1A.

## Introduction

Discovered in 1965, adeno associated viruses (AAVs) have become a cornerstone system for delivering gene therapies for several inherited diseases. AAVs are small viruses (20–25 nm in diameter) comprising a 60-protein subunit capsid surrounding a single-stranded, ∼4.7 kb DNA genome flanked by inverted terminal repeats (ITRs).^1^^,^^2^ Importantly, AAVs are non-pathogenic in humans, and AAV vectors are now used as gene delivery vehicles in 7 gene therapies approved by the US Food and Drug Administration (FDA) since 2007. All currently approved AAV-based gene therapies utilize gene replacement for treating recessive disorders (https://www.fda.gov/vaccines-blood-biologics/cellular-gene-therapy-products/approved-cellular-and-gene-therapy-products). However, gene replacement is not indicated for dominant diseases, many of which would likely benefit from disease gene silencing. Indeed, we previously published several pre-clinical AAV-based gene therapy approaches to treat various dominant disorders, including Charcot-Marie-Tooth disease type 1A (CMT1A) and Ffcioscapulohumeral muscular dystrophy (FSHD).^3^^,^^4^^,^^5^^,^^6^^,^^7^^,^^8^^,^^9^^,^^10^^,^^11^^,^^12^ In each program, we use AAVs to deliver DNA cassettes expressing artificial miRNAs engineered to silence dominant disease genes, including *PMP22* for CMT1A and *DUX4* for FSHD.^9^^,^^11^^,^^12^

Clinical translation and potential commercialization of these therapies will require development of a battery of conformance tests to ensure consistent product quality and gain regulatory approval. Specifically, according to the US Code of Federal Regulations (21 CFR 610.1), “no lot of any licensed product shall be released by the manufacturer prior to the completion of tests for conformity with standards applicable to such product.” For AAV products, conformance tests measure critical attributes, such as capsid and genome identity, purity, stability, safety/sterility, and potency. In this study, we focused on methodology for *in vitro* potency assay development to support CMT1A and FSHD gene therapies.

A potency assay is used to ensure lot-to-lot consistency in AAV manufacturing and, thus, also plays a valuable role in comparability studies and stability testing.^13^ During therapeutic development, prior to clinical application, a potency assay is a valuable tool to assess AAV manufacturing consistency or variances that could impact the strength of the final product. As such, regulatory agencies recommend designing an appropriate potency assay as soon as possible, to ensure product potency across the entire drug development pipeline.^13^ Potency is defined by the FDA as “the specific ability or capacity of the product to effect a given result” (21 CFR 600.3(s)).^14^ The CMT1A and FSHD gene therapy products described here affect a given result by silencing the *PMP22* or *DUX4* mRNAs, respectively, following delivery by AAV vectors.^9^^,^^11^^,^^12^ Mechanistically, vectors for both programs carry engineered miRNA expression cassettes (miR871 for CMT1A and mi405 for FSHD), driven by the U6 promoter, designed to trigger RNA interference (RNAi) against the *PMP22* or *DUX4* mRNA. Silencing of *PMP22* or *DUX4* mRNA leads to reduction of toxic PMP22 or DUX4 protein levels in Schwann cells (CMT1A) or skeletal muscle (FSHD).^9^^,^^11^^,^^12^

Potency measurements may be relatively straightforward for some therapies, for example, using cell-free methods to determine binding affinity of therapeutic antibodies to a desired target.^15^ In contrast, gene therapy potency assay development is arguably more complex because it requires a biological system and involves a multi-step process to produce a quantifiable therapeutic effect inside a target cell. Specifically, AAV vectors must first bind a host cell, translocate across the cell membrane, escape the *trans*-Golgi network, transport to the nucleus, and uncoat the therapeutic DNA genome to allow host transcriptional machinery to produce the desired therapeutic gene product.^16^ Thus, gene therapies cannot be adequately tested in a cell-free system. Although FDA guidance states that *in vivo* potency assays using animal models are acceptable for gene therapy programs, *in vitro* assays that limit the use of animals are encouraged.^13^^,^^17^ In this study, we aimed to create versatile and easy-to-implement *in vitro*, cell-based potency assays for gene silencing therapies. The cells and assays described should be applicable for measuring potency of any viral or non-viral gene silencing product targeting *PMP22* or *DUX4* mRNA.

## Results

### Production and clonal isolation of stable cell lines for potency assay development

**Figure 1:**
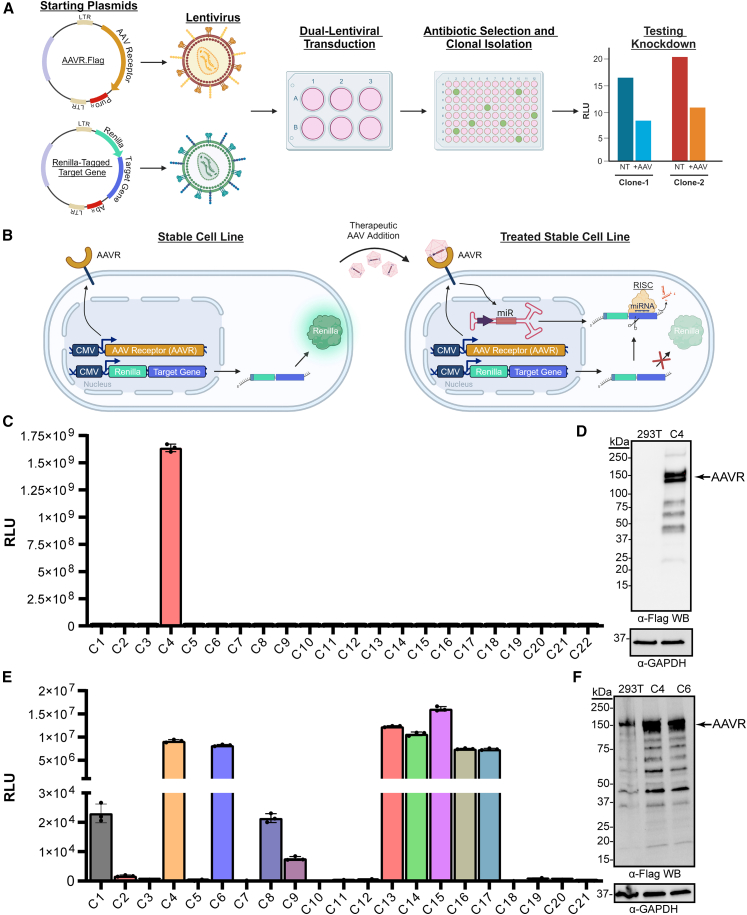
Stable cell-line generation, design, and screen for *rLuc* and AAVR.flag expression (A) Flow chart of stable cell line generation. Top plasmid, AAVR.flag-PuroR. Bottom plasmid, schematic represents either rLuc^PMP22^-NeoR or rLuc^DUX4^-BlastR. AbR, antibiotic resistance gene, where AbR is neomycin resistance (NeoR) or blasticidin resistance (blastR) as indicated. Cells were produced using dual lentiviral transduction on a 6-well plate followed by antibiotic selection and single-cell isolation. Clones were tested for AAVR.flag expression, improved AAV transduction, and *rLuc* knockdown after treatment. (B) Schematic of resulting cell line and potency assay from (A). Stable cell line with AAVR for improved transduction, and *rLuc*^*PMP22/DUX4*^. After AAV treatment, miRNAs are transcribed in the host cell nucleus, processed by RNAi machinery, and loaded into the RNA-induced silencing complex (RISC) to trigger target transcript degradation and *rLuc* signal reduction. (C) Relative light units (RLU) produced from 40,000 cells of 22 different CMT1A clones plated in a 96-well format. Only one NeoR/PuroR-resistant CMT1A line (clone C4^CMT1A^) produced *Renilla* luciferase expression. (D) Clone C4^CMT1A^ expresses the FLAG-tagged AAVR protein. Image shows a western blot of protein lysates from clone C4^CMT1A^ or control HEK293T cells, probed with an HRP-conjugated anti-FLAG antibody. AAVR has a predicted molecular weight of 108 kDa, but due to glycosylation, migrates at ∼150 kDa, as seen here. The blot was then stripped and probed with a rabbit anti-GAPDH antibody. (E) RLU produced from 40,000 cells of 21 different FSHD clones plated in a 96-well format. 11 of 21 clones showed luciferase expression above background. (F) Clones C4^FSHD^ and C6^FSHD^ over-express the AAVR protein, as shown by western blot, similar to (D). Bar graphs display mean (SD) using experimental and biological triplicates (*N* = 3 biological replicates with each condition performed in triplicate).

Our strategy for potency assay development required creation of AAV-permissive stable cell lines capable of producing quantifiable and reproducible readouts of target gene knockdown using a simple reporter assay. In this study, we generated three such cell lines—one for CMT1A and two for FSHD—each using similar methods. We began with HEK293T cells because they are easily passaged and produce robust RNAi-mediated gene silencing effects, making them useful for testing the mechanism of action of our CMT1A and FSHD gene silencing products. Unfortunately, HEK293T cells are not readily transduced by many AAV serotypes, and we hypothesized that AAV transduction could be improved by integrating KIAA0319L, which encodes a protein also known as the universal AAV receptor (AAVR).^18^ In addition, we inserted *rLuc* reporter genes containing *PMP22* or *DUX4* cDNA sequences (*rLuc*^*PMP22*^ or *rLuc*^*DUX4*^) within the 3′ UTR to enable quantification of miR871- or mi405-mediated *PMP22* or *DUX4* gene silencing, respectively (Figure 1).

To create stable cell lines harboring both AAVR and *rLuc*^*PMP22*^ or *rLuc*^*DUX4*^, we generated 3 different lentiviral vectors and performed dual lentiviral transduction in HEK293T cells (Figure 1A). Specifically, one lentiviral vector delivered a FLAG-tagged AAVR cDNA (AAVR.flag) co-expressed with a puromycin resistance (Puro^R^) gene for selection. The other two lentiviral vectors carried *rLuc*^*PMP22*^ or *rLuc*^*DUX4*^ co-expressing a neomycin resistance (Neo^R^) or blasticidin resistance (Blast^R^) gene, respectively. Cells co-transduced with AAVR and *rLuc*^*PMP22*^ lentiviral vectors were selected using puromycin and G418 whereas those treated with AAVR and *rLuc*^*DUX4*^ were selected with puromycin and blasticidin. Surviving polyclonal cells were seeded on 96-well plates at a concentration of 0.5 cells/well to enable clonal isolation. Single-cell clones were confirmed using microscopy and expanded to generate a cryopreserved cell bank. Clones were then tested to confirm they possessed both AAVR.flag and the *rLuc-*tagged target gene (Figures 1C–1F).

### Stable cell line clones contain AAVR.flag and *rLuc* signal

We first created the AAVR/*rLuc*^*PMP22*^ cell line utilizing a neomycin resistance gene and G418 selection in HEK293T cells. We identified 22 surviving AAVR/*rLuc*^*PMP22*^ G418-resistant single-cell clones, expanded each and seeded 40,000 cells per clone on a 96-well plate. Of the 22 initial isolates, only one expressed a significant amount of *rLuc* (clone C4) (Figure 1C). This low percentage of luciferase-positive clones likely resulted from reduced selection, since HEK293T cells already contain a neomycin resistance gene. Importantly, in addition to expressing *rLuc* , we used an anti-FLAG western blot to confirm that clone C4 had robust AAVR.flag expression (Figure 1D). Henceforth, we refer to this stable cell line for CMT1A potency assay development as C4^CMT1A^.

To improve the success rate for creating a stable cell line for an FSHD potency assay, we changed the antibiotic resistance gene on the *rLuc*^*DUX4*^ lentiviral vector from Neo^R^ to Blast^R^ and selected clones using blasticidin. This improved selection method yielded 10 of 21 clones expressing *rLuc*^*DUX4*^ (Figure 1E). To identify clones that exhibited dose-dependent *rLuc*^*DUX4*^ knockdown, we treated each cell line with AAV9 or AAV.SLB101, an engineered myotropic capsid developed by Solid Biosciences, each containing an identical AAV genome expressing our therapeutic miRNA targeting *DUX4* (mi405) (Figure S1). While several clones showed AAV.mi405-mediated silencing of *rLuc*^*DUX4*^, we selected two leads, named C4^FSHD^ and C6^FSHD^, based on robust AAVR expression as determined by western blot (Figure S1; Figure 1F).

### Stable AAVR-expressing cell lines improve transduction efficiency by multiple AAV serotypes

**Figure 2:**
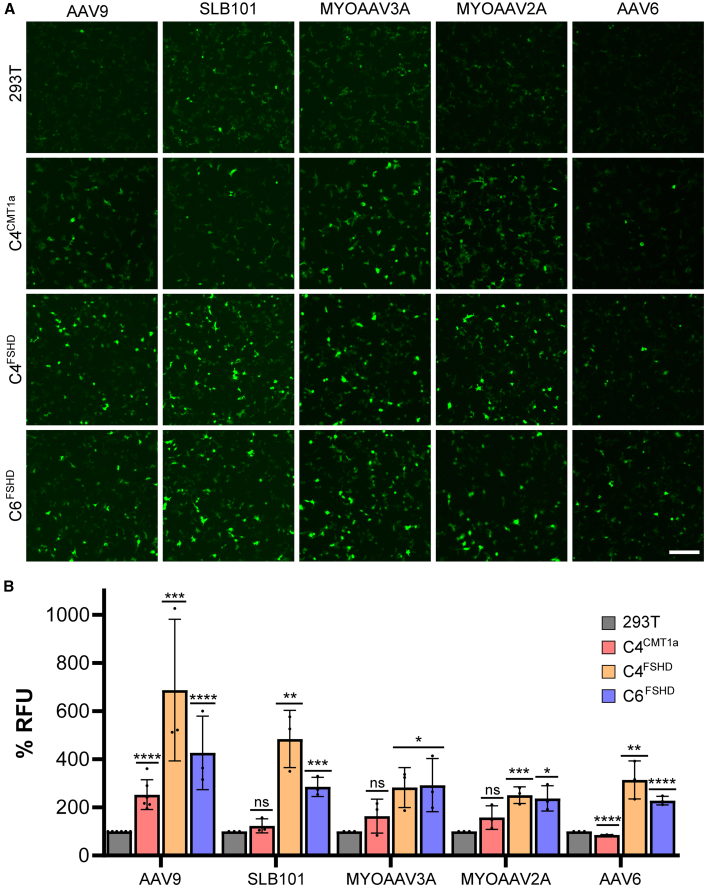
Assessment of AAV vector transduction in C4^CMT1A^, C4^FSHD^, and C6^FSHD^ cell lines 10,000 cells were plated in a 96-well format and transduced by AAV9, SLB101, MYOAAV3A, MYOAAV2A, or AAV6 expressing CMV.GFP genomes. (A) Fluorescence microscopy images demonstrate visual extent of transduction after 24 h. White scale bar representing 250 μm in the bottom right of C6^FSHD^ treated with AAV6 CMV.GFP (B) Quantification of GFP signal 48 h after transduction using a GloMax plate reader and plotted as % RFU (relative fluorescent units) compared to untransduced HEK293T cells. Data represent mean (SD), with significance determined using non-parametric *t* test vs. 293T cells, ∗∗∗∗*p* < 0.0001, ∗∗∗*p* < 0.005, ∗∗*p* < 0.01, ∗*p* < 0.05, ns *p* > 0.05. (*N* = 3 biological replicates with each condition performed in triplicate).

We confirmed our hypothesis that stable AAVR over-expression would improve transduction efficiency of multiple AAV serotypes. To do this, we transduced C4^CMT1A^, C4^FSHD^, and C6^FSHD^ cell lines with five AAV serotypes, 2 naturally occurring (AAV9 and AAV6) and 3 engineered (Solid Bio's POLARIS-101, referred to here as SLB101, MYOAAV3A, and MYOAAV2A), all expressing a CMV.GFP construct.^19^^,^^20^^,^^21^^,^^22^^,^^23^ We first qualitatively assessed transduction efficiency 24 h after AAV treatment using fluorescence microscopy imaging (Figure 2A). We then quantified GFP expression using a GloMax plate reader at 48 h (Figure 2B).

Compared to parent HEK293T cells, AAV9 transduction efficiency was significantly improved for all three clones tested. Surprisingly, the three engineered serotypes, SLB101, MYOAAV3A, and MYOAAV2A, had significantly improved transduction on C4^FSHD^ and C6^FSHD^ but not C4^CMT1A^ cells. However, C4^FSHD^ and C6^FSHD^ were in part selected by screening clones using the myotropic SLB101 capsid, which could explain the differences in permissibility between FSHD and CMT1A cell lines (Figure S1). In addition, AAV6 transduction was reduced in C4^CMT1A^ cells but significantly improved in both FSHD cell lines, although at roughly half the levels achieved by SLB101. Importantly, the improved tropism of AAV9 for C4^CMT1A^ and SLB101 for both C4^FSHD^ and C6^FSHD^ cell lines supports our translational programs, as we intend to use AAV9 to deliver U6.miR871 to CMT1A Schwann cells and SLB101 to deliver U6.mi405 to FSHD skeletal muscle. We performed a second transduction experiment to further demonstrate that lentiviral vector insertion of AAVR could improve AAV permissibility in HEK293Ts (Figure S2). Specifically, we transduced 100,000 C4^CMT1A^, C4^FSHD^, and C6^FSHD^ cells with AAV9.miR871 at a multiplicity of infection (MOI) of 2.67E6 and then measured AAV DNA genomes from cell lysates by ddPCR 24 h later. Although the miR871 does not target the *DUX4* sequence in the FSHD cell lines, we selected the AAV9.miR871 vector for this experiment because AAV9 transduction was universally increased in all 3 cell lines based on the GFP experiment (Figure 2). In agreement with the GFP fluorescence data, all stable cell lines showed significantly increased intracellular AAV DNA genomes (plotted as vector genomes/nanogram DNA; vg/ng) compared to HEK293T cells (Figure S2). Together, the GFP and miR871 ddPCR data demonstrated that lentiviral insertion of AAVR improved AAV transduction, as expected.

### Therapeutic AAV potency measurements using stable cell lines

**Figure 3:**
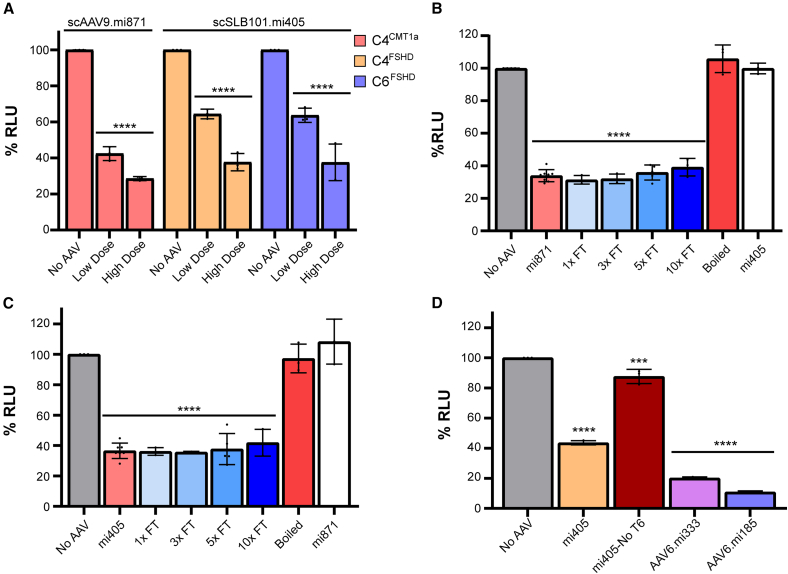
Stable cell lines are useful for CMT1A and FSHD gene therapy potency assays *(*A) Dose-dependent, treatment-specific knockdown of rLuc activity in C4^CMT1A^, C4^FSHD^, and C6^FSHD^ stable cells treated with two doses of therapeutic AAV. For CMT1A, scAAV9.mi871; for FSHD, SLB101.mi405. (B and C) Potency assay identifies defective or non-functional AAV vectors. C4^CMT1A^ and C4^FSHD^ treated with indicated vectors with and without multiple cycles of freeze-thaw (1×, 3×, 5×, and 10×) or boiling (98°C for 5 min). SLB101.mi405 served as negative control for C4^CMT1A^ cells and AAV9.miR871 served as negative control for C4^FSHD^ cells. Lack of rLuc knockdown by negative controls in both assays supports assay specificity. (D) C4^FSHD^ cells detect *DUX4*-targeted silencing from multiple miRNAs. C4^FSHD^ cells were transduced by AAV6 vectors expressing mi405, a control mi405 lacking a terminator sequence (mi405-No T6), or two additional vectors expressing artificial miRNAs that bind and silence *DUX4* at different locations (mi333 and mi185). RLU values represent mean (SD), with significance determined using one-way ANOVA, ∗∗∗∗*p* < 0.0001, ∗∗∗*p* < 0.005, ∗∗*p* < 0.01, ns *p* > 0.05.

An ideal potency assay is robust, reproducible, and quantifiable. To assess if our cell lines can be used to measure potency, we treated each with indication-relevant therapeutic AAVs, or controls, and measured the *rLuc*^*PMP*^ or *rLuc*^*DUX4*^ signal 24 or 48 h later, respectively. For the CMT1A potency assay, we treated C4^CMT1A^ cells with the AAV9.miR871 vector at low (9E5) and high (8E6) doses. For the FSHD potency assay, we treated C4^FSHD^ and C6^FSHD^ cells with low (1.6E6) and high (8E6) doses of SLB101.mi405 vector. Both vectors produced dose-dependent *rLuc* reductions in their respective cell lines, relative to untreated cells (Figure 3A). Specifically, for CMT1A, our therapeutic AAV9.miR871 vector significantly reduced the *rLuc*^*PMP22*^ signal in C4^CMT1A^ cells by 57% (low dose) and 78% (high dose) (Figure 3A). Similarly, both FSHD cell lines, C4^FSHD^ and C6^FSHD^, showed nearly identical dose-dependent *rLuc*^*DUX4*^ signal reduction of ∼36% (low dose) and ∼64% (high dose). To ensure the reduced *rLuc* signal was not due to decreased viability or cell number following transduction, we performed an ATPase cell titer assay following AAV transduction at 3 different doses of AAV9.miR871 (8E6, 2.67E6, and 9E5 MOI for C4^CMT1A^) or SLB101.mi405 (8E6, 1.6E6, and 3.2E5 MOI for C4^FSHD^ and C6^FSHD^). We observed little to no impact on cell viability and used these data to further refine the parameters of the potency assay (Table S1). Specifically, we propose to use reference standard vectors to ensure assay consistency, based on FDA guidelines; as such, for CMT1A potency assays, we propose to use a quality-controlled AAV9.miR871 reference standard vector at 8E6 MOI on C4^CMT1A^ cells and a quality-controlled SLB101.mi405 reference standard vector at 1.6E6 MOI on C4^FSHD^ cells.^24^

We also tested additional quality control metrics, including the linearity of the rLuc signal across an 8-step serial dilution of each cell line, and assessed any potential changes in the rLuc signal with increasing passage number (Figure 3). To this point in the study, we used 10,000 cells per well on a 24-well plate for each potency assay. To determine the dynamic range of rLuc activity as a function of cell number, we performed serial dilutions of C4^CMT1A^, C4^FSHD^, and C6^FSHD^ cells. Specifically, we plated cells at densities of 313, 625, 1.25k, 2.5k, 5k, 10k, 20k, and 40k, and measured rLuc activity 24 h later. All 3 lines showed tight linearity of signal in this cell density range (C4^CMT1A^, R^2^ = 0.997; C4^FSHD^, R2 = 0.983; C6^FSHD^, R2 = 0.976), with robust signal (in relative light units; RLU) exceeding 1E6 raw units even at the lowest cell density (313 cells per 24-well plate) (Table S1). In addition, because the CMV promoter used to drive expression of our AAVR and rLuc has been previously suggested to be susceptible to epigenetic silencing over time, we assessed rLuc activity at different cell passages. Because we typically do not grow HEK293T cells past passage 30, we measured luciferase activity in C4^CMT1A^ cells at passages 20, 23, 28, and then in a newly thawed plug of cells at passage 2. In a similar experiment, we measured luciferase activity in C4^FSHD^ cells at passages 15, 17, 26, and then in a newly thawed plug of cells at passage 2. We measured similar levels of RLU among the untreated cell passages (Figures S3C and S3E). We also treated the same cells at the same passages with AAV9.miR871 (for CMT1A) or SLB101.mi405 (for FSHD) at 8E6 MOI and observed similar levels of luciferase reporter knockdown in all conditions (Figure S3). These results suggested that promoter silencing is not occurring at the cell passage numbers we intend to use for these assays. We therefore conclude that our approach to dispose of cells before passage 30 will sufficiently ensure that CMV promoter silencing is not occurring.

To demonstrate assay specificity, we used additional controls including treated cells with inactivated therapeutic vectors and an AAV expressing a non-targeting miRNA for the relevant target gene in each assay. To inactivate AAV9.miR871 and SLB101.mi405 vectors, we subjected each to 1, 3, 5, or 10 freeze-thaw cycles from −80°C to 37°C, and separately heat-treated (boiled) a second set of vectors at 98°C for 5 min. For CMT1A, we used an MOI of 8E6 to treat C4^CMT1A^ cells with (1) therapeutic AAV9.miR871; (2) 4 different freeze-thawed samples of AAV9.miR871; (3) heat-inactivated AAV9.miR871; and (4) to remain consistent with serotype, AAV9.mi405, which targets *DUX4* and not the *PMP22* sequences present in the *rLuc*^*PMP22*^ transcript. Compared to untreated C4^CMT1A^ cells, AAV9.miR871-treated cells showed a significant 66% reduction in *rLuc*^*PMP*^^22^ activity. Interestingly, up to 10 freeze-thaw cycles had no impact on AAV9.miR871 potency in this assay, as freeze-thawed AAV9.miR871 samples still triggered *rLuc*^*PMP22*^ knockdown to levels produced by fresh AAV9.miR871 (Figure 3B). In contrast, the boiled AAV9.miR871 and the AAV9.mi405 control failed to reduce *rLuc*^*PMP22*^ activity in C4^CMT1A^ cells. We performed a similar set of experiments for the FSHD potency assay, using an 8E6 MOI of active SLB101.mi405, freeze-thawed SLB101.mi405, and heat-inactivated SLB101.mi405 in C4^FSHD^ cells. For the non-targeting control, we used an AAV9.miR871 vector; whereas, the control serotype is different (AAV9 vs. SLB101), we demonstrated that AAV9 and SLB101 both efficiently transduce FSHD cell lines (Figure 2B). The results in the FSHD assay were similar to those produced in the CMT1A assay (Figure 3B–C). Specifically, neat and freeze-thawed SLB101.mi405 significantly reduced *rLuc*^*DUX4*^ signal to similar levels (∼60%), while heat-inactivated SLB101.mi405 and non-targeting AAV9.miR871 had no impact on *rLuc*^*DUX4*^ activity compared to untreated control C4^FSHD^ cells (Figure 3C).

Finally, to demonstrate the universality of our potency assay strategy, we transduced C4^FSHD^ cells with the same dose (8E6 MOI) of SLB101.mi405; an SLB101 vector containing a control mi405 lacking a T6 terminator (mi405-No T6); and 2 other previously published *DUX4*-targeting miRNAs, mi333 and mi185, each packaged in AAV6, and both of which had been stored at 4°C for several years prior to use (Figure 3D).^12^ The SLB101.mi405 vector again silenced *rLuc*^*DUX4*^ by ∼60% while the absence of a terminator sequence on mi405 only reduced the *rLuc*^*DUX4*^ by ∼10%. The other miRNA candidates, mi333 and mi185, significantly silenced *rLuc*^*DUX4*^ by 80%–90%, demonstrating the assay could be used to assess potency of *DUX4*-targeting sequences beyond mi405 (Figure 3D).

## Discussion

Nearly 30 viral and non-viral *in vivo* gene therapy products are now FDA-approved for use in the United States, with various analysts predicting more than 60 additional approved products by 2030.^25^^,^^26^ Rapidly evolving gene therapy technology, such as engineered AAV capsids or development of safer, *in vivo* gene-editing approaches, could also expand the field by providing tools to treat disease targets for which gene therapy was not previously indicated. Prior to clinical use, each program will require development of a potency assay, which typically needs to be uniquely designed on a drug-by-drug basis, potentially costing valuable time and resources. In this study, we sought to improve the efficiency of potency assay development for gene silencing therapies using dual lentiviral transduction to generate AAV-permissible stable cell lines and a modular, gene-specific, quantifiable luciferase readout of product potency (i.e., gene silencing). We propose that this platform approach will expedite potency assay development and regulatory approval for viral and non-viral gene silencing therapies.

To ensure simplicity and avoid complications of using animal models, we developed a HEK293T cell based *in vitro* potency assay. To improve the typically poor AAV transduction in HEK293T cells, we stably integrated the universal AAVR using lentiviral vectors, and selected 3 cell lines (1 for CMT1A, 2 for FSHD) to further test AAV transduction (Figures 1 and 2). We found variable transduction of different serotypes among the 3 lines, despite using the same AAVR-expressing lentiviral vector at the same MOI (Figure 2). For example, although AAV9 transduction was significantly improved in all 3 stable cell lines (C4^CMT1A^, C4^FSHD^, and C6^FSHD^), it was highest in FSHD cells compared to the single CMT1A clone. In contrast, AAVR integration significantly improved SLB101 transduction of C4^FSHD^ and C6^FSHD^ lines but had no positive impact on the C4^CMT1A^ clone, which remained largely non-permissive to SLB101 (Figure 2). We hypothesize that stochastic insertion of the AAVR lentiviral vector at different locations within the HEK293T clones could account for the differences but did not map the number or location of AAVR integration. Nevertheless, the clones we generated were relevant for the respective programs we are developing. Specifically, the C4^CMT1A^ clone was permissive to the AAV9 vector used in our CMT1A gene therapy program (AAV9.miR871), and similarly, the two FSHD lines (C4^FSHD^ and C6^FSHD^) were permissive to the SLB101 capsid we intend to use in our FSHD program (SLB101.mi405). These results underscore the importance of screening program-relevant serotypes after AAVR lentiviral vector delivery and using appropriate antibiotic selection to produce more clones for transduction screening.

The second key feature of our potency assay involved integrating a reporter gene to provide a simple and quantitative readout of *PMP22* or *DUX4* gene silencing. To do this, we inserted the *PMP22* or *DUX4* open-reading frames as the 3′ UTR of *rLuc*, creating a fusion mRNA instead of a fusion protein. This design prevented any potential toxicity that could arise from PMP22 or DUX4 protein expression, while also providing mRNA-binding sites for our engineered miRNAs, thereby allowing quantification of *PMP22* or *DUX4* gene knockdown in a dose-dependent manner following AAV treatment or using any gene-silencing/gene-editing modality. This approach can be adapted by replacing *PMP22* or *DUX4* with any gene sequence in the *rLuc* 3′ UTR, if the target sequence meets lentiviral packaging requirements.

One theoretical technical issue with our system has to do with the use of the CMV promoter to drive transcription of the AAVR and the reporter genes in our stable clones. Specifically, the CMV promoter may be epigenetically silenced under some conditions, which could potentially render our potency assays non-functional over time. However, CMV promoter silencing is not universally found in every cell line and has been reported to be strongly dependent on transgene insertion site.^27^^,^^28^ To date, we have observed no significant reduction in luciferase signal up to passage ∼30 from any of our stable lines nor comparing living versus cryopreserved cells (Figure S3). Nevertheless, to avoid transgene silencing, we propose to dispose of cells prior to passage 30 and use low-passage cell banks to replenish potency assay stocks. Should transgene silencing occur, we could treat cells with 5-aza-cytidine to potentially reverse epigenetic silencing of the CMV promoter. In addition, to ensure potency assay reliability, we propose to follow FDA guidelines and use a reference virus that has already passed QC and would serve as a positive control.^17^^,^^24^ If any drift in *rLuc* signal or AAVR expression occurs, it could be detected based on the control virus knockdown compared to historical data. Ultimately, the purpose of a potency assay is to compare a new clinical batch of vector to a previous batch to ensure manufacturing consistency, and inclusion of a control vector would account for intra- and inter-assay variability.

In summary, we propose that our potency assay approach is valuable because it provides a framework to rapidly develop this critical assay early in therapeutic development, as recommended by the FDA. This provides many advantages, primarily the ability to assess how manufacturing or storage conditions alter product strength prior to additional *in vivo* or bridging studies. In addition, unlike more standardized methods for product identity or purity (e.g., determining empty, full, or partial capsids), a potency assay needs to be developed for every new therapeutic target. In short, streamlining the process for potency assays will help reduce the developmental burden when designing and testing new therapeutic products. As such, this methodology may aid in expediting regulatory approval of gene therapies targeting dominant diseases.

## Materials and methods

All materials and methods are available in the supplemental information file.

## Data and code availability

All unique reagents generated in this study are available upon request.

## Acknowledgments

The authors wish to thank Solid Biosciences for providing the engineered AAV capsid POLARIS-101™ (SLB101) and Dr. Tatyana Vetter for assistance with microscopy. This work was funded by Armatus Bio under a sponsored research agreement to S.Q.H. Graphical abstract and Figure 1 created in BioRender by J.M. (2026) https://BioRender.com/5lg389d and https://BioRender.com/13r6ugc.

## Author contributions

Conceptualization, J.M., L.M.W., B.P., R.S., and S.Q.H.; formal analysis, J.M.; funding acquisition, B.P., R.S., and S.Q.H.; investigation, J.M. and B.Z.; methodology, J.M., B.Z., L.M.W., and S.Q.H.; project administration, J.M. and S.Q.H.; supervision, J.M. and S.Q.H.; validation, J.M. and B.Z.; visualization of published work, J.M.; writing, J.M. and S.Q.H. All authors read, edited, and approved the final submitted manuscript.

## Declaration of interests

B.P., R.S., L.M.W., and S.Q.H. are all paid consultants or employees of Armatus Bio, a private biotech company that funded this study. B.P., R.S., and S.Q.H. are all shareholders in Armatus Bio. A provisional U.S. patent application has been filed on this technology (application #63/716,014, “Products and methods for measuring the potency of a silencing gene therapy”, with J.M., L.M.W., B.P., R.S., and S.Q.H. listed as inventors.

## Supplemental information

Document S1. Figures S1–S3 and Tables S1

Document S2. Article plus supplemental information
