# Supplementary material for: Development of in vitro potency assays for AAV-based gene silencing therapies targeting FSHD and CMT1A
Source: Mol Ther Adv. 2026 Apr 1;34(2):201727. doi: 10.1016/j.omta.2026.201727 (PMC13148907; doi:10.1016/j.omta.2026.201727)
Supplement: Document S1. Figures S1–S3 and Tables S1 [file mmc1.pdf]

OMTA, Volume *34*

Supplemental information

Development of *in vitro* potency assays for AAV-based gene silencing therapies targeting FSHD and CMT1A

Jason McCoy, Lindsay M. Wallace, Bi Zhou, Brian Price, Rachel Salzman, and Scott Q. Harper

MATERIALS AND METHODS

Generation of proviral lentiviral plasmids and lentiviral vector production

The AAVR.flag lentiviral plasmid was acquired from Addgene (#166716) and used to generate lentiviral vectors (LVs). To generate $C4^{CMT1A}$ cells, we replaced GFP in the pLenti-CMV-GFP-Neo plasmid (Addgene, #17447) with $rLuc^{PMP22}$ using XbaI and SalI restriction enzyme sites. The $rLuc^{PMP22}$ cDNA was first generated by inserting the full-length human *PMP22* open reading frame and 3' UTR within the psiCHECK2 dual luciferase vector (Promega). This construct was inserted after the rLuc stop codon, thereby yielding a fusion mRNA in which *PMP22* serves as the rLuc 3' UTR. Then, $rLuc^{PMP22}$ DNA was PCR amplified to add 5' AvrII and 3' SalI restriction sites and ligated into the same sites on the pLenti backbone using DNA ligation (Takara; #6023) per manufacturer's instructions. To generate raw materials to create FSHD cell lines, we inserted a PCR-amplified $rLuc^{DUX4}$ cDNA containing 5' SalI and 3' XbaI restriction sites and cloned this fragment into pLenti-CMV-Blast-empty (Addgene, #17468) digested with the same enzymes. $rLuc^{DUX4}$ was PCR-amplified from a previously described plasmid[8]. The pLenti-$rLuc^{PMP22}$-Neo and pLenti-$rLuc^{DUX4}$-Blast plasmids were sequence-confirmed prior to lentiviral production. All three lentiviral 77 were made by Genewiz from Azenta Life Sciences.

Stable HEK293T cell production and clonal isolation

We generated three VSVG-pseudotyped lentiviral vectors carrying the following constructs: (1) flag-tagged AAVR (AAVR.flag) co-expressing a puromycin resistance ($Puro^R$) gene; (2) $rLuc^{PMP22}$ co-expressing a neomycin resistance (Neo^R) gene; and (3) $rLuc^{DUX4}$ co-expressing a blasticidin resistance gene ($Blast^R$). Stable HEK293T cells were generated using dual lentiviral transduction, with one vector expressing AAVR.flag and a second expressing gene-relevant luciferase reporters ($rLuc^{PMP22}$ for CMT1A cells, or AAVR.flag and $rLuc^{DUX4}$ for FSHD cells). HEK293T cells were co-transduced with the described vectors at MOI of 5, 2.5, 1.25, 0.625, or 0.325, with an additional untransduced well serving as a selection indicator. Each LV was diluted in 500 μl of Dulbecco's

Modified Eagle Medium (DMEM) + 10mg/mL polybrene to the indicated MOI and added to individual wells on a 6-well plate. A reverse transduction was conducted by adding 1 mL of DMEM+10mg/mL polybrene containing 50,000 HEK293T cells to each well. The 6-well plate was incubated at 37º C, 5% CO_2 for 72hrs prior to adding selection antibiotics. CMT1A cell lines were selected using puromycin (2 µg/mL) and G418 (600 µg/mL). FSHD cell lines were selected using puromycin (0.5 µg/mL) and blasticidin (2.5 µg/mL). Media was replaced every 48-72 hours until all cells in the control well were dead and transduced cells reached 50-60% confluency. Transduced cells were transferred to a 10 cm plate for further expansion and single cell isolation.

Single cell isolation

Transduced cells were diluted to a concentration of 5 cells/mL in a 50/50 mix of 0.22 µm filtered conditioned media (CM) from untransduced HEK293T cells and DMEM + 1% FBS. 100 µl of diluted cells were seeded to each well of a 96-well plate. Plates were then incubated undisturbed for one week at 37º C, 5% CO_2. Wells with surviving single colonies were expanded to 20-40% confluency, then transferred to a 24-well plate. After clones reached 50-60% confluency on a 24-well plate they were transferred to a 6-well plate, until once again reaching 50-60% confluency, at which point they were transferred to a 10 cm plate. The 10 cm plate was then used for further experiments, passaging, and generating a liquid nitrogen stock.

***Renilla* luciferase assay**

To detect *Renilla* luciferase signal in stable cell line clones, 40,000 cells were plated in triplicate wells of a 96-well plate. We then used a *Renilla* luciferase assay kit (Promega catalog number E2820) per manufacturer's instructions. In short, 2-4 hours after cells attached to the plate, growth media was removed and replaced with 1x lysis buffer from the Promega kit and incubated with gentle agitation for 20-30 minutes. After lysis, the 96-well plate was placed in a GloMax plate reader. The GloMax injector dispensed 100 µl of 1x *Renilla* substrate per well followed by a 2

second hold and 10 second integration, with data reported in relative light units (RLU). The RLU of each well was then graphed using GraphPad Prism 10.

Anti-flag western blot

Five million CMT1A or FSHD stable cells were pelleted at 500 x g for 5 minutes and lysed with 1 mL ice-cold RIPA buffer (Thermo Scientific, catalog #89901) for 30 minutes. Lysates were centrifuged at 16,000 x g for 10 minutes, and then 5 μl added to reducing Laemmli SDS sample buffer with 2-betamercaptoethanol (BME) followed by electrophoresis using a 4-20% Mini-PROTEAN® TGX™ precast protein gel (BioRad, Catalog number 4561094). Samples were transferred onto a PVDF membrane using the Trans-Blot turbo transfer system and RTA transfer kit (BioRad, Catalog number 1704274). Membranes were then blocked using 1% dehydrated milk in TBS-T and treated with horseradish peroxidase (HRP)-coupled anti-flag (α-Flag.HRP) antibody (Proteintech, catalog number HRP-66008) at a 1:15,000 dilution. The blot was then washed and imaged with Immobilon™ western chemiluminescent HRP substrate (ECL Cat: WBKLS0500) using a ChemiDoc-MP by BioRad. Western blot membranes were then stripped for 30 minutes at room temperature on an orbital shaker using Restore™ western blot stripping buffer (Thermo Scientific, catalog number 21059). Membranes were then re-blocked and treated with anti-GAPDH antibody (Abcam, catalog number ab181602) at a 1:10,000 dilution for 1 hour at room temperature. The blots were washed and treated with an HRP-coupled goat anti-rabbit antibody (Jackson Laboratories catalog number 111-035-144) at a 1:20,000 dilution for 2 hours at room temperature. The blot was then washed and imaged with Immobilon™ western chemiluminescent HRP substrate (ECL Cat: WBKLS0500) using a ChemiDoc-MP by BioRad.

AAV Production

All AAV vectors used in this study were produced by Andelyn Biosciences (Columbus, Ohio), with three exceptions. SLB101.mi405-No T6 was produced at the University of Massachusetts viral vector core (Worcester, Massachusetts), while AAV6.mi333 and AAV6.mi185 were produced in

the Harper lab using a standard AAV triple transfection production protocol in HEK293 cells. In short, 48 hrs after transfection of plasmids expressing AAV2 *rep* and AAV6 *cap* genes, adenovirus helper genes (pHelper), and AAV proviral constructs carrying U6.mi405-No T6, U6.mi333, or U6.mi185, cells were lysed using 3x freeze thaw cycles and cellular debris pelleted by centrifugation. Cell lysates were then subjected to iodixanol density gradient ultracentrifugation and fast protein liquid chromatography (FPLC) to purify the AAV. Vector yields were determined by QPCR or ddPCR using primer/probe sets detecting the AAV2 ITR sequences.

AAV.GFP transduction imaging

10,000 unmodified or stable HEK293T cells were seeded on a 96-well plate and allowed to adhere for 2-4 hrs. Cells were then transduced with 5 different AAV serotypes containing a CMV.GFP genome: AAV9, AAV6, MYOAAV3A, and MYOAAV2A (multiplicity of infection, MOI: 2.67E6) and SLB101 (MOI: 1.6E6). 24 hours later, wells were qualitatively assessed using fluorescent microscopy. To quantify GFP expression at 48 hrs, media was replaced with 50 μl PBS and total green fluorescence measured using a Promega GlowMax instrument. RFU was normalized to unmodified HEK293T cells and percent RFU (%RFU) plotted using GraphPad Prism 10.

Potency assay using *Renilla* luciferase as outcome measure

To test the potency of AAV vectors, 10,000 cells of each stable cell line were seeded on a 96-well plate. Following a 2 hr adherence period, cells were treated with therapeutic AAV vectors at an MOI of 9E5 (low dose) or 8E6 (high dose) for CMT1A cell lines, and MOI of 1.6E6 (low dose) and 8E6 (high dose) for FSHD cell lines. The rLuc signal was measured 24 hrs later for CMT1A cell lines and 48 hrs later for FSHD cell lines, using Promega's *Renilla* Luciferase Assay System (Cat: E2820). In short, media was removed and replaced with 30 µl of 1x *Renilla* Luciferase Assay Lysis Buffer and placed on an orbital shaker for 15-20 minutes. Lysates were then transferred to a black bottom, black walled 96 well plate (Millipore, catalog number MSSBNFX40) and placed in a GlowMax instrument. *Renilla* luciferase signal was measured one well at a time using the

following instrument protocol parameters: 100 µL of *Renilla* Luciferase Assay Reagent was added to a well followed by a 2 second hold, and luminescence was then read at an interval of 0.3 seconds for a total of 10 seconds. All measurements were then integrated and reported as a single RLU value. RLU was normalized to untransduced stable cells and plotted using GraphPad Prism 10.

Droplet digital PCR (ddPCR)

To determine transduction efficiency of AAV9.miR871 on generated clones ($C4^{CMT1A}$, $C4^{FSHD}$, $C6^{FSHD}$) and parent HEK293T cell controls, 100,000 cells of each line were seeded on a 24-well plate. Cells were placed in a 37º C, 4% CO_2 incubator for 2 hours to adhere to the plate. Cells were then treated with AAV9.miR871 at an MOI of 2.67E6 and placed back into the incubator for 24 hours. After 24 hours growth media was removed, cells were washed with 1 mL of PBS, and lifted from the plate with 500 µl of Trypsin-EDTA (0.05%) (Thermo Scientific catalog number 25300062) for 5 minutes. Trypsinized cells were harvested and centrifuged at 300 x g for 5 minutes, and then supernatant was removed and resuspended in 200 µl PBS. DNA was then purified using Qiagen's DNeasy® Blood & Tissue Kit (catalog number 69504) per manufacturer's instructions. DNA was quantified using a Qubit™ dsDNA high sensitivity (HS) assay per manufacturer's instructions. Droplet digital PCR was conducted using BioRad's ddPCR supermix for probes (no dUTP) (catalog number 1863024) and the following primer-probe set ordered from Integrated DNA Technologies (IDT):

Fwd Primer: 5'-CGGCCTCAGTGAGCGA- 3'

Rev Primer: 5' -GGAACCCCTAGTGATGGAGTT- 3'

FAM probe: 5'-/56-FMA/CACTCCCTCTCTGCGCGCTCG /3BHQ_1/-3'

Extracted DNA was diluted and, 0.0715 ng, 0.0143 ng, and 0.00286 ng was added to the PCR mixture. Droplets were generated using the Bio-Rad automated droplet generator per manufacturer instruction. Droplets were then subjected to a two-step PCR reaction:

Step 1: 95º C, 10 s

Step 2: 94º C, 30 s

Sep 3: 60º C, 1 minute

Repeat Steps 2 + 3, 39 cycles

Step 4: 98º C, 10 minutes

Step 5: Infinite hold 12º C

The FAM signal was then measured using the Bio-Rad QX200 droplet reader and data processed using QX Manager Standard Edition. The copies/ul readout was then normalized to input DNA to determine the vg/ng plotted using GraphPad Prism.

CellTiter-Glo assay

To conduct the CellTiter-Glo® assay by Promega (catalog number: G7571), cells were treated just as they were for the potency assay using *Renilla* luciferase. 10,000 cells of each stable cell line were seeded on a 96-well plate. Following a 2-hr adherence period, cells were treated with therapeutic AAV vectors at an MOI of 9E5, 2.67E6 and 8E6 for CMT1A cell lines, and MOI of 3.25E6, 1.6E6, and 8E6 for FSHD cell lines. The CellTiter-Glow® assay was conducted 24hrs later for CMT1A cell lines and 48hrs later for FSHD cell lines, using. In short, plates were equilibrated to room temperature for 30minutes, then, 100µl of the CellTiter-Glo® reagent was added to each treatment well and an empty control well and placed on an orbital shaker for 10-15 minutes. Lysates were then transferred to a white bottom, white walled 96 well plate (Promega, catalog

number E5650) and placed in a GlowMax instrument. Luminescent signal was measured one well at a time using an integration time of 0.3s. RLU was normalized to untransduced stable cells and plotted using GraphPad Prism 10.

Serial dilution of stable cell lines for *rLuc* linearity

Stable cell lines were counted and seeded in a 96-well plate using a 1:2 serial dilution from 40,000 to 313 cells, including: 40,000, 20,000, 10,000, 5,000, 2,500, 1,250, 625, and 313 cells per well. Following a 2-3 hr adherence period, media was removed and rLuc signal measured using Promega's *Renilla* Luciferase Assay System (Cat: E2820). In short, media was removed and replaced with 30 μl of 1x *Renilla* Luciferase Assay Lysis Buffer and placed on an orbital shaker for 15-20 minutes. Lysates were then transferred to black bottom, black walled 96-well plates (Millipore, catalog number MSSBNFX40) and placed in a GlowMax instrument. *Renilla* luciferase signal was measured one well at a time using the following instrument protocol parameters: 100 μL of *Renilla* Luciferase Assay Reagent was added to a well followed by a 2 second hold, and luminescence was then read at an interval of 0.3 seconds for a total of 10 seconds. All measurements were then integrated and reported as a single RLU value. Raw RLU values and cell count were log transformed and plotted using GraphPad Prism 10 and fit using simple linear regression.

Table S1: CellTiter-Glo® Viability Assay. Data reported as mean (SD) % viability normalized to untreated cells.

**Cell Line**	**8E6 MOI**	**2.67E6 MOI**	**9E5 MOI**
$C4^{CMT1A}$	98.2% (±1.17%)	96.7% (±1.28%)	96.5% (±1.50%)
	**8E6 MOI**	**1.6E6 MOI**	**3.2E5 MOI**
$C4^{FSHD}$	95.5% (±1.49%)	101% (±1.72%)	102% (±2.17%)
$C6^{FSHD}$	93.7% (±1.43%)	99.6% (±1.69%)	103% (±2.03%)

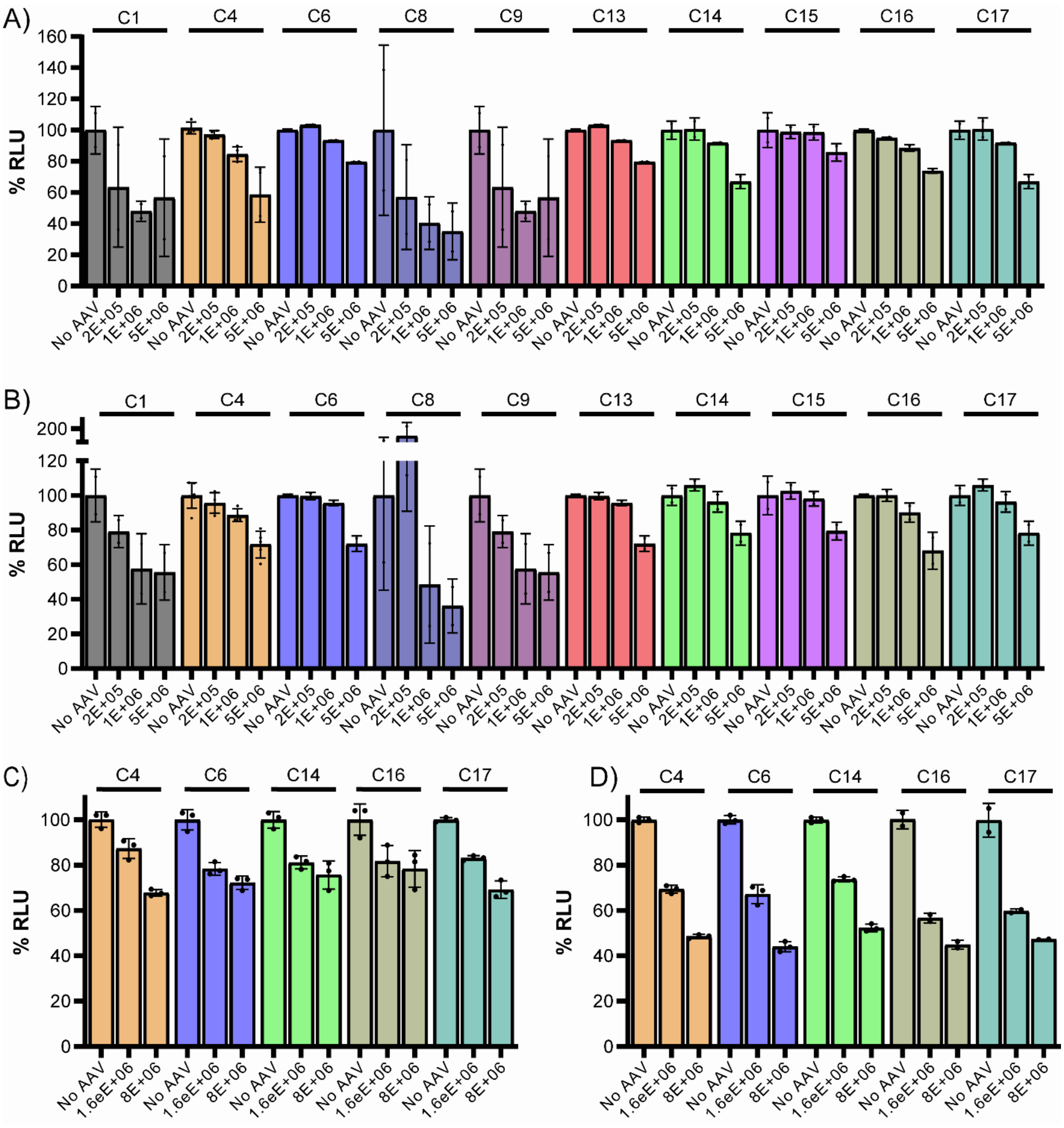

Figure S1. Screening FSHD stable cell lines for mi405-mediated silencing. (**A-B**) Testing dose-dependent knockdown of rLuc^{DUX4} activity in FSHD cell lines 24 hrs following treatment with (A) scAAV9.mi405 or (B) SLB101.mi405, using indicated multiplicities of infection (MOI), normalized to untreated cells (No AAV). The top 5 candidates in A-B were then confirmed in a second round

of experiments. **(C-D)** rLuc^{DUX4} knockdown 24 hrs (C) or 48 hrs (D) after AAV9.mi405 treatment with MOI of 8E6 and 1.6E6 per well, compared to untreated cells (No AAV). C4 and C6 clones were selected for additional testing due to significant dose response 48 hrs after SLB101.mi405 treatment. Preliminary screen only conducted once to narrow down candidate with experimental triplicate and plotted as mean (SD) using GraphPad10.

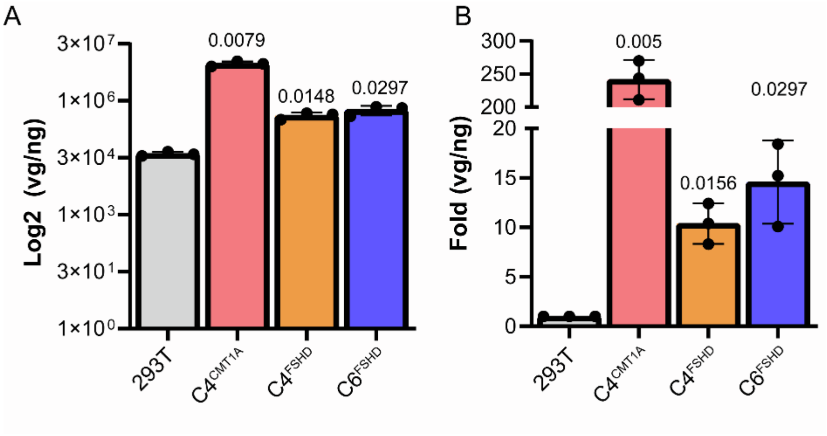

Figure S2. Stable, clonal insertion of AAVR in 3 different cell lines improves AAV9.miR871 transduction compared to parent HEK293T cells. **(A)** AAV DNA genomes measured by droplet digital PCR (ddPCR) 24 hours after adding 2.67E6 MOI of AAV9.miR871 to indicated wells. Data plotted as log_2-transformed vector genomes per nanogram of DNA (vg/ng). **(B)** Same data in (A) plotted as fold-change compared to control HEK293T cells lacking lentiviral insertion of AAVR. Data represent means +/- standard deviation (SD), with significance determined using Welch's t-test of clonal cell lines versus parent HEK293T cells and resulting p-value placed above each bar. (N=3 biological replicates with at least 2 dilutions within range using ddPCR).

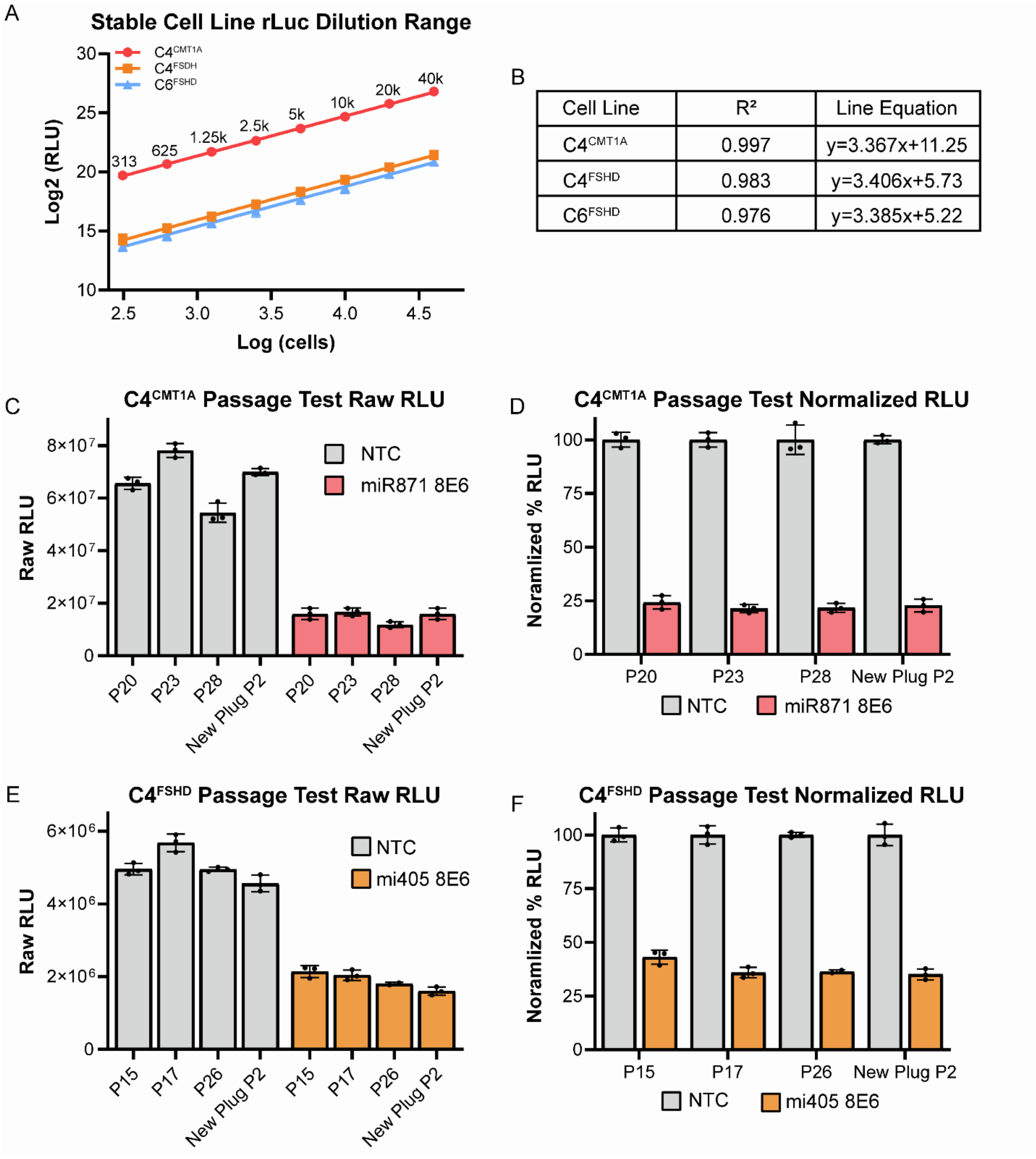

Figure S3. Assessment of *Renilla* luciferase signal dynamic range and durability during stable cell line passaging. (A) *Renilla* luciferase signal ($rLuc^{DUX4}$ for FSHD; $rLuc^{PMP22}$ for CMT1A lines) showed linearity across an 8-step serial dilution (313, 625, 1.25K, 2.5K, 5K, 10K, 20K, and 40K). Graph plots log(cells) vs log2(RLU). **(B)** Simple linear-regression R^2 values and line

equation generated from data in A. **(C)** Raw RLU values from C4^{CMT1A} cells treated with AAV9.miR871 (MOI of 8E6) at 4 indicated passages, or left untransduced (NTC, non-transduced control). **(D)** RLU values from (C) normalized to untreated C4^{CMT1A} controls. **(E)** Raw RLU values from C4^{FSHD} cells treated with SLB101.mi405 (MOI of 8E6) at 4 indicated passages, or left untransduced. **(F)** RLU values from (E) normalized to untreated C4^{FSHD} controls. Bar graphs display mean +/- standard deviation (SD) from N=3 experiments performed in triplicate (A) or N=1 experiments performed in triplicate (C-F).
